# The strong correlation between *ADAM33* expression and airway inflammation in chronic obstructive pulmonary disease and candidate for biomarker and treatment of COPD

**DOI:** 10.1038/s41598-021-02615-2

**Published:** 2021-11-30

**Authors:** Muhammad Fachri, Mochammad Hatta, Muhammad Nasrum Massi, Arif Santoso, Tri Ariguntar Wikanningtyas, Ressy Dwiyanti, Ade Rifka Junita, Muhammad Reza Primaguna, Muhammad Sabir

**Affiliations:** 1 Department of Pulmonology and Respiratory Medicine, Faculty of Medicine and Health, Universitas Muhammadiyah, Jakarta, Indonesia; 2 Molecular Biology and Immunology Laboratory, Faculty of Medicine, Hasanuddin University, Makassar, Indonesia; 3 Department of Pulmonology and Respiratory Medicine, Faculty of Medicine, Hasanuddin University, Makassar, Indonesia; 4 Department of Clinical Pathology, Faculty of Medicine and Health, Universitas Muhammadiyah, Jakarta, Indonesia; 5 Department of Medical Microbiology, Faculty of Medicine, Tadulako University, Palu, Indonesia; 6 Department of Internal Medicine, Faculty of Medicine, Hasanuddin University, Makassar, Indonesia

**Keywords:** Genetics research, Translational research

## Abstract

Airway inflammation in patients with chronic obstructive pulmonary disease (COPD) is an amplified response of the normal immune system that occurs as a result of chronic irritation by toxic substances, such as cigarette smoke. This leads to the characteristic pathological changes in the inflammatory cells of COPD patients. *ADAM33* has been reported to be involved in the pathogenesis of COPD in East Asia by affecting airway inflammation and other immune responses. The aim of this study was to determine the potential role of *ADAM33* (mRNA and soluble levels) as a biomarker of inflammation in COPD patients. This is a case control study using consecutive sampling. The COPD case and control (non-COPD) groups comprised 37 and 29 patients, respectively. We used univariate analysis to assess differences in the parameters between the groups and bivariate analysis to non-parametrically compare these parameters between the two groups. We observed significantly higher mRNA levels of *ADAM33* in the COPD patients (10.39 ± 1.76) as compared to that in the non-COPD individuals (6.93 ± 0.39; P < 0.001). The levels of soluble *ADAM33* were also significantly higher in the COPD patients (2.188 ± 1.142 ng/ml) compared to the non-COPD individuals (0.487 ± 0.105 ng/ml; P < 0.001). The mRNA and soluble *ADAM33* levels were significantly higher in COPD patients compared to those in the parameter-matched non-COPD individuals. Thus, *ADAM33* is a potential biomarker and treatment for inflammation in COPD patients.

## Introduction

Chronic obstructive pulmonary disease (COPD) is a chronic lung disease that manifests with persistent respiratory symptoms and expiratory airflow limitation. Exposure to harmful particles or toxic gases results in abnormalities in the airways or alveoli that lead to COPD^1,2^. Airway inflammation in COPD patients is an amplified inflammatory response to chronic irritation, such as cigarette smoke. Among several studies, Wang et al. reported the involvement of *ADAM33* in the pathogenesis of COPD in the East Asian population: *ADAM33* regulates airway inflammation and immune response in COPD patients. Laxmi et al. showed a significant correlation between the genetic polymorphisms in *ADAM33* S1-A/G and S2-C/G and pathophysiology of COPD in the South Indian population. The inflammatory response associated with COPD includes changes in pro-inflammatory or anti-inflammatory cytokines and occurs predominantly in the presence of *ADAM33* polymorphisms^3–5^.

Kim et al. demonstrated that active vitamin D_3_ regulates vascular endothelial growth factor (VEGF) that stimulates *ADAM33* expression and proliferation in smooth muscle cells; this can be used to influence the treatment outcome in patients with asthma^6^. Zhang et al. showed that lipopolysaccharide decreases the proliferation and functionality of human primary lung fibroblasts (important targets in patients with COPD)^7^. Increased mRNA and soluble levels of *ADAM33* can serve as bridges for these cytokines that results in damage to the matrix. In this study, we investigated the role of *ADAM33* (mRNA and soluble form) in matrix damage as part of the pathomechanism involved in COPD.

## Methods

### Study design and population

This is a case control study using consecutive sampling. The COPD case group included 37 patients, and the control group consisted of 29 non-COPD patients who were healthy people living near the hospital. Inclusion Criteria The control group (non-COPD) is non-COPD patients aged over 40 years who live in the vicinity of the Pondok Kopi and Sukapura Islamic Hospitals in Jakarta and are willing to voluntarily participate in the entire research program by signing an informed consent form. Exclusion Criteria Control group (non-COPD), i.e not COPD patients with other lung diseases (e.g.: asthma, pulmonary TB, pneumonia and lung tumors). The inclusion criteria for the subject group of COPD patients are stable COPD patients aged over 40 years who come to the pulmonary polyclinic of the Jakarta Islamic Hospital Pondok Kopi and Sukapura and are willing to voluntarily participate in the entire research program by signing an informed consent form. Exclusion Criteria Subject group COPD patients, namely COPD patients with other lung diseases (e.g.: asthma, pulmonary TB, pneumonia and lung tumors). We used IBM SPSS Statistics version 23 in this study. Univariate analysis was used to evaluate the differences in the parameters between groups. Bivariate analysis was performed to compare these parameters between two groups by non-parametric statistics. The study was performed in accordance with the ethical standards of the Declaration of Helsinki (1964) and its subsequent amendments.

### COPD patients

COPD is a preventable and treatable chronic lung disease characterized by persistent respiratory symptoms and airflow limitation due to airway and/or alveolar abnormalities usually caused by exposure to noxious particles or noxious gases^1,2^.

The diagnosis of COPD was based on the Global Initiative for Chronic Obstructive Lung Disease (GOLD) criteria:

Stage I: mild, FEV_1_/FVC < 70%, FEV_1_ ≥ 80% predicted (post bronchodilator test).

Stage II: moderate, FEV_1_/FVC < 70%, 50% < FEV_1_ < 80% predicted (post bronchodilator test).

Stage III: severe, FEV_1_/FVC < 70%, 30% < FEV_1_ ≤ 50% predicted (post bronchodilator test).

Stage IV: very severe, FEV_1_/FVC < 70%, FEV_1_ ≤ 30% predicted (post bronchodilator test).

Chronic Obstructive Pulmonary Disease (COPD) in this study were all COPD patients with at least Stage I GOLD COPD. The diagnosis of COPD was based on lung function in the Global Initiative for Chronic Obstructive Lung Disease (GOLD) criteria. Meanwhile, the non-COPD control group had lung function within normal limits^1,2^.

### *ADAM33* mRNA levels

The mRNA levels of *ADAM33* were determined by extracting nucleic acids using the method described in previous study^8,9^. Briefly, ~ 100 µl of peripheral blood was incubated with 900 μl of a solution consisting of guanidium thiocyanate. Subsequently, a 20-μl suspension consisting of 50 ml of H_2_O and 500 μl of 32% (w/v) diatom was added to the solution. We removed the supernatant and washed the sediment with a buffer followed by two washes with 1 ml of 70% ethanol and 1 ml of acetone. The resulting solution was heated in a water bath at 56 °C for 10 min and added to 60 μl of TE buffer consisting of 1 mM EDTA in 10 mM Tris HCl (pH 8.0). The supernatant from this mixture was transferred into a fresh tube for nucleic acid isolation and stored at − 80 °C until subjected to polymerase chain reaction (PCR)^8,9^.

The expression profile of target genes was determined using real-time PCR (qRT-PCR). Expression was represented as a ratio of expression of the primary oligonucleotide-specific gene to that of *GAPDH* (housekeeping gene). *ADAM33* mRNA was detected using specific forward (5′-CAGGAATGCCAGCTATTATC-3′) and reverse (5′-GTTTGGTGTGGTTCAAGTTT-3′) primers. *GAPDH* was detected using specific forward (5′-GGCCAAAAGGGTCATCATC-3′) and reverse (5′-GTGATGGCATGGACTGTGG-3′) primers. The PCR thermal protocol was as follows: for *ADAM33*, 38 cycles of 94 °C for 3 min and 54 °C for 30 s; for *GAPDH*, 32 cycles of 94 °C for 10 s and 54 °C for 30 s according to the protocol described by Kim et al.^6^. qRT-PCR was performed with the One-Step SYBR Green qRT-PCR kit optimized for the Real-Time PCR CFX 6400 thermal cycler [6.10]. The total reaction volume was 25 µl (including experimental RNA) with 12.5 µl of the 2 × SYBR Green qRT-PCR master mix and “x” µl of the concentration-optimized primer stocks. Subsequently, “x” µl of nuclease-free water along with concentration-optimized final primers, 0.375 µl of the reference dye solution from stage 1 (optional), and 1 µl of the RT/RNase block enzyme mixture were added to a total reaction volume of 50 µl. The reaction was mixed slowly to avoid frothing (without rotating) and “x” µl of the RNA-solution mixture was added to individual experimental PCR tubes. The reaction was mixed slowly, briefly centrifuged, and placed in the instrument. The PCR program was run using a real-time PCR machine (CFX Connect system, Bio-Rad Laboratories, Real-Time PCR, 96 wells, 0.1 ml, USA)^6,9–11^.

### Levels of soluble *ADAM33*, Interleukin (IL)-6, IL-8, IL-10, matrix metalloproteinase (MMP)-9

Levels of soluble *ADAM33*, IL-6, IL-8, IL-10 and MMP-9 were measured by enzyme-linked immunosorbent assay (ELISA). Patient serum samples were prepared using an *ADAM33*, IL-6, IL-8, IL-10 and MMP-9 kit at room temperature. Each sample was analyzed in duplicates to ensure the validity of the data obtained by ELISA. Initially, 100 µl of the Assay Diluent-containing protein buffer was added to each well. Next, 100 µl of Standard fluid-containing recombinant human target from a predetermined kit or diluted patient serum samples (1:10) was added to each well. The plate was then incubated for 2 h at room temperature. The liquid was removed and each well was washed four times with sterile phosphate-buffered saline. Then, 200 µl of Conjugate buffer with horseradish peroxidase-streptavidin was added to each well, the plate was covered with a plastic lid, and incubated at room temperature for 2 h. The liquid was removed and plate was washed four times with sterile phosphate-buffered saline. Next, 200 µl of Substrate Solution containing TMB was added to each well. The plate was incubated at room temperature for 20 min in the dark. After incubation, the reaction was stopped by adding 50 µl of Stop Solution containing H_2_SO_4_ to each well following which levels were measured within 30 min using the ELISA Reader 270 (Biomerieux, France) at a wavelength of 450 nm. The target soluble protein concentration was represented in ng/ml^7,12–14^.

### Ethics approval and consent to participate

This research was submitted to the ethics committee of the Faculty of Medicine, Hasanuddin University, Makassar, Indonesia (No. 1006/H4.8.4.5.31/PP36-KOMETIK/2017, November 27, 2017) to obtain approval for ethical studies. Written informed consent was obtained from all participants.

## Results

Table 1 shows that there were more males with COPD as compared to females with COPD. Patients with COPD were between 60 and 80 years and were presented to our hospital at an average age of 65.68 years. In comparison, the non-COPD patient controls were between 60 and 81 years with an average age of 67 years. The highest Brinkmann index (3) was found in patients with COPD and non-smokers (0) in non-COPD control individuals. There were no differences in patient characteristics between groups (P > 0.05). Individuals in both groups were homogeneous based on sex, age, and Brinkmann index.

**Table 1:** Basic characteristics of COPD patients and non-COPD individuals.

Patient characteristics	Group				P value
	COPD (cases; n = 37)		Non-COPD (Control; n = 29)		
**Gender**					
Male	33	89.19%	25	86.20%	0.471
Female	4	10.81%	4	13.79%	
Age (years old): mean (SD)/min–max	65.68 (5.40)/60–80		67 (6.63)/60–81		0.269
**Brinkmann Index**					
0	5		17		0.973
1	6		4		
2	9		6		
3	17		2		

This study also looked at the characteristics of the COPD patient group which was divided based on COPD patients based on the stage of GOLD obstruction, based on the value of post bronchodilator obstruction can be seen in the summary analysis in the table below showing the highest number of COPD obstruction stages GOLD II with a total of 12 COPD patients (32.43%). in the Table 2 below is shown in full.

**Table 2:** Characteristics of COPD patients stage of obstruction GOLD.

COPD obstruction stage GOLD	Amount	Percentage (%)
I	10	27.03
II	12	32.43
III	10	27.03
IV	5	13.51

**Figure 1:**
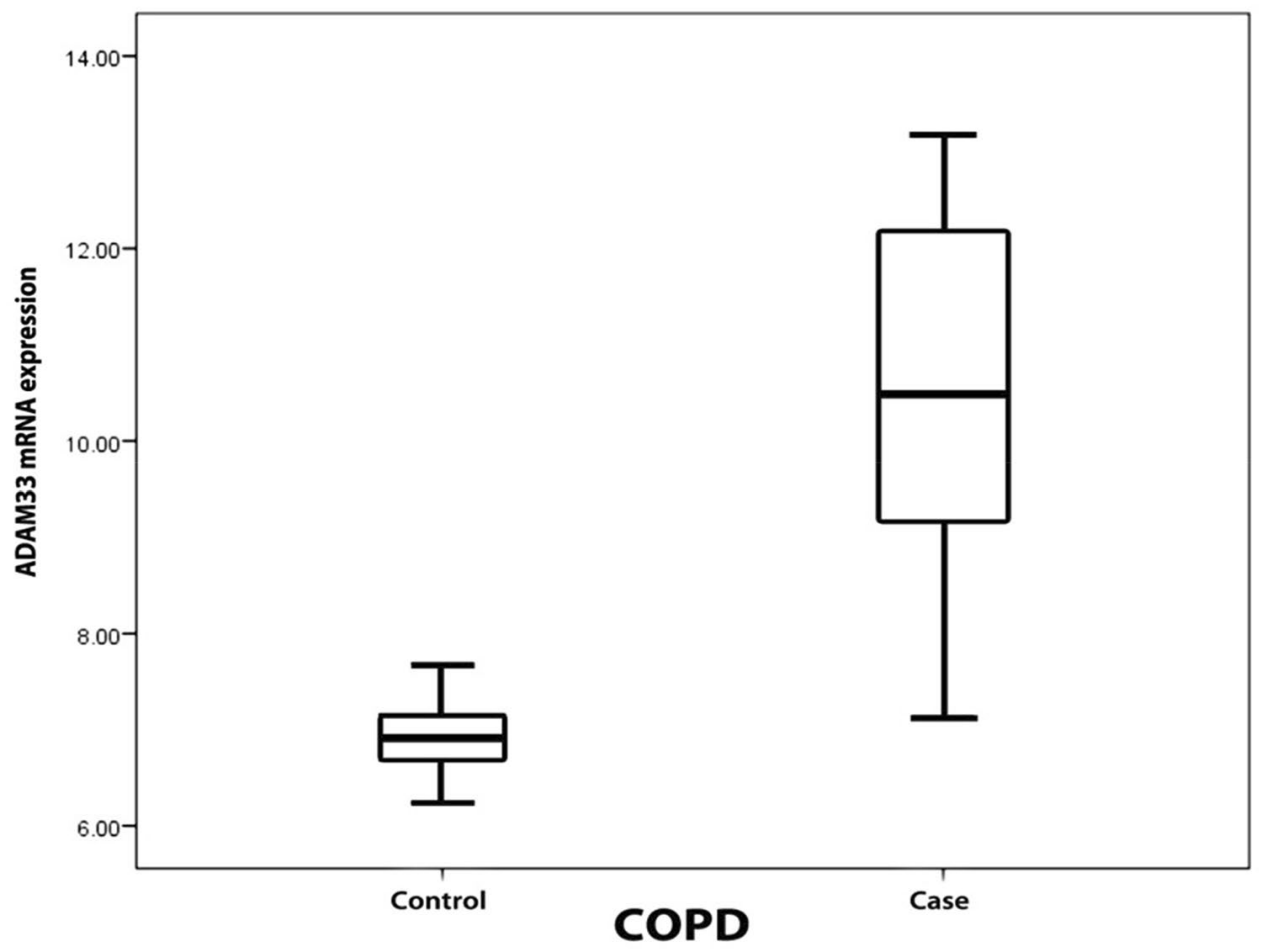
Boxplot for the levels of *ADAM33* mRNA in COPD patients and non-COPD individuals.

**Figure 2:**
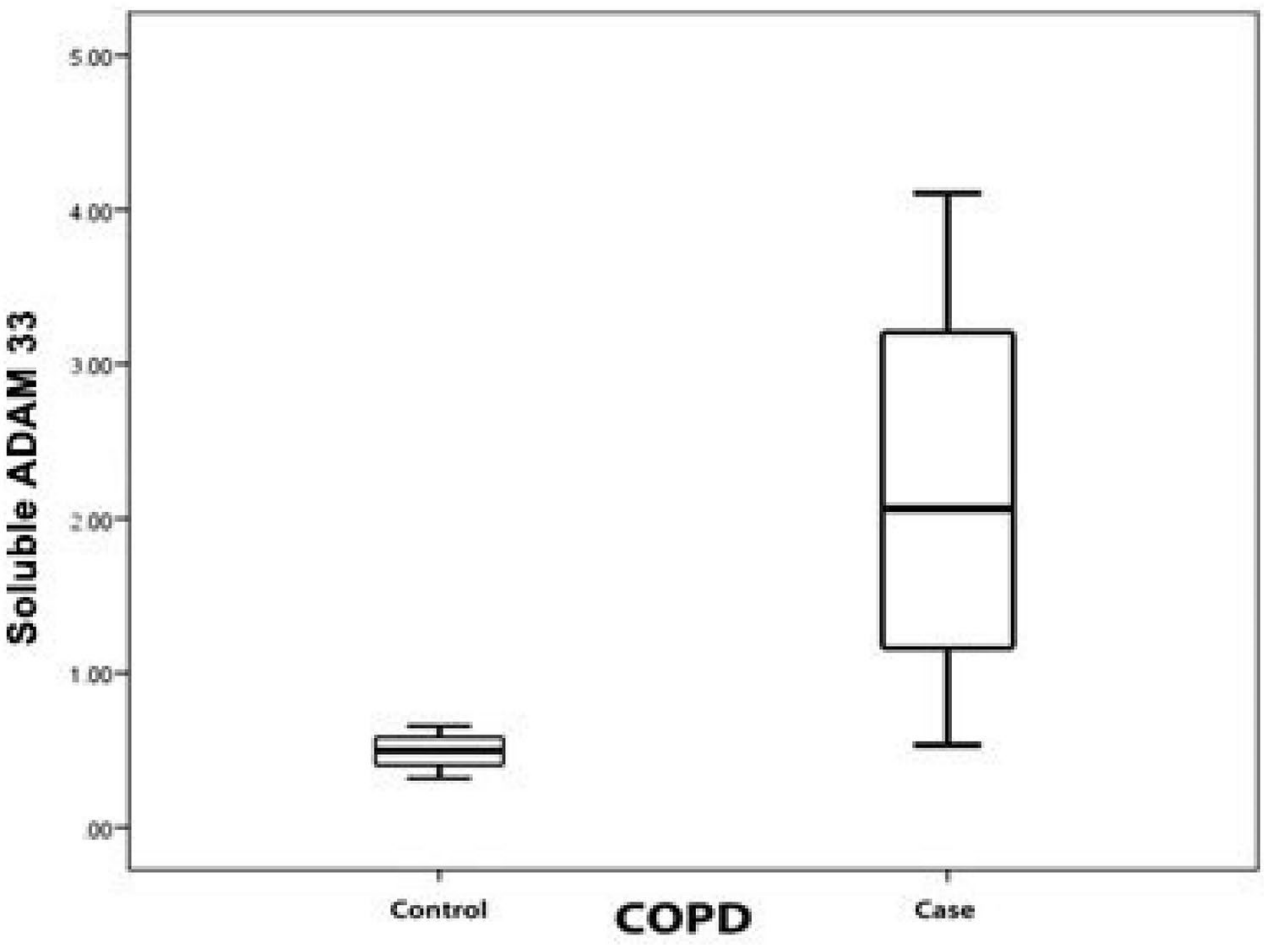
Boxplot for the levels of soluble *ADAM33* in COPD patients and non-COPD individuals.

The mRNA levels of *ADAM33* were significantly higher in COPD patients (10.39 ± 1.76 fold change; 95% confidence interval (CI) 9.802–10.981) as compared to that in non-COPD individuals (6.93 ± 0.39 fold change; 95% CI 6.780–7.079; P < 0.001; Fig. 1). Similarly, the levels of soluble *ADAM33* were also significantly higher in COPD patients (2.188 ± 1.142 ng/ml; 95% CI 1.807–2.569) as compared to that in non-COPD individuals (0.487 ± 0.105 ng/ml; 95% CI 0.447–0.526; P < 0.001; Fig. 2).

The role of pro-inflammatory cytokines (IL-6 and IL-8) and anti-inflammatory cytokines (IL-10) in COPD patients with differences in soluble IL-6 and IL-8 levels being higher in COPD patients than in non-COPD patients, while Lower IL-10 in COPD patients than in non-COPD patients; each of which found a statistically significant difference (P < 0.01). This is shown in the Table 3 below.

**Table 3:** Differences in soluble levels of IL-6, IL-8, IL-10 in COPD patients and non COPD patients.

Biomarkers	Not a COPD patient (n = 29)		COPD patient(n = 37)		P*
	Mean (SD)	95% CI	Mean (SD)	95% CI	
Soluble IL-6 (pg/ml)	2.619 (0.5840)	2.397–2.842	25.526 (18.866)	19.90–31.15	< 0.001
Soluble IL-8 (pg/ml)	133.63 (33.386)	120.93–146.33	513.457 (331.796)	402.83–624.084	< 0.001
Soluble IL-10 (pg/ml)	311.23 (8.12)	308.1–314.3	240.01 (112.34)	202.6–277.5	0.001

*Independent sample t test.

Matrix damage in COPD patients was found to be consistent with the presence of increased levels of soluble MMP-9, the matrix marker studied in this study was MMP-9. Table 4 below shows that there is matrix damage in COPD patients with differences in soluble MMP-9 levels being higher in COPD patients than in non-COPD patients; which were statistically significant (P < 0.01).

**Table 4:** Differences in soluble levels of MMP-9 in COPD patients and non-COPD patients.

Biomarker	Not a COPD patient (n = 29)		COPD patient (n = 37)		P*
	Mean (SD)	95% CI	Mean (SD)	95% CI	
Soluble MMP-9 (ng/ml)	2.105 (0.16)	2.043–2.165	5.205 (2.126)	4.496–5.913	< 0.001

*Independent sample t test.

Factors associated with *ADAM33* that have changed in COPD patients: MMP-9 and Cytokines (IL-6, IL-8 and IL-10). Table 5 below shows that there is a significant linear correlation between soluble *ADAM33* levels and *ADAM33* mRNA expression with soluble MMP-9 levels in COPD patients through the Spearman Correlation test (P < 0.001), so the higher the soluble *ADAM33* level, the higher the soluble MMP-9 level. Serum levels, as well as the expression of *ADAM33* mRNA on soluble MMP-9 levels. However, there was no correlation between soluble *ADAM33* levels and *ADAM33* mRNA expression with soluble MMP-9 levels in non-COPD patients (P > 0.05). This proves that there is a relationship between *ADAM33* and MMP-9 in COPD pathomechanism.

**Table 5:** Correlation of ADAM33 Soluble Levels and ADAM33 mRNA expression with MMP-9 in COPD patients and non-COPD patients.

Variable correlation	Case (COPD patient) (n = 37)		Control (not COPD PATIENT) (n = 29)	
	r	P*	R	P*
Soluble ADAM33 with soluble MMP-9	0.967	< 0.001	− 0.015	0.470
ADAM33 mRNA expression with soluble MMP-9	0.899	< 0.001	0.078	0.345

*Spearman correlation.

This study also showed that there was a significant linear correlation, levels of soluble cytokines IL-6, IL-8 and IL-10 and mRNA expression of the *ADAM33* gene with soluble *ADAM33* levels in COPD patients through the Spearman Correlation test (P < 0.001). And there was no correlation between levels of soluble cytokines IL-6, IL-8, IL-10 and ADAM33 gene mRNA expression with soluble *ADAM33* levels in non-COPD patients (P > 0.05). As shown in Table 6 below, it proves that there is a relationship between IL-6, IL-8 and IL-10 cytokines with *ADAM33* in COPD pathomechanism.

**Table 6:** Correlation between soluble cytokines levels (IL-6, IL-8 dan IL-10) and ADAM33

Variable correlation	Case (COPD patient) (n = 37)		Control (not COPD patient) (n = 29)	
	R	P*	R	P*
Soluble ADAM33 with soluble IL-6	0.976	< 0.001	− 0.186	0.167
Soluble ADAM33 with soluble IL-8	0.976	< 0.001	− 0.211	0.138
Soluble ADAM33 with soluble IL-10	− 0.947	< 0.001	− 0.106	0.292
Soluble ADAM33 with ADAM33 mRNA expression	0.897	< 0.001	− 0.032	0.435
Soluble IL-6 with ADAM33 mRNA expression	0.945	< 0.001	− 0.079	0.343
Soluble IL-8 with ADAM33 mRNA expression	0.908	< 0.001	− 0.228	0.118
Soluble IL-10 with ADAM33 mRNA expression	− 0.950	< 0.001	− 0.076	0.347

*Spearman correlation.

This study also showed that there was a linear correlation in the direction of soluble cytokine levels IL-6, IL-8 and IL-10 with soluble MMP-9 levels which was significant in COPD patients through the Spearman Correlation test (P < 0.001). There was also a correlation between soluble cytokine levels IL-8 and IL-10 with soluble MMP-9 levels in non-COPD patients (P < 0.001). And there was no correlation between the levels of soluble cytokine IL-6 with soluble levels of MMP-9 in non-COPD patients (P > 0.05). As in Table 7 below, it explains that there is a relationship between IL-6, IL-8 and IL-10 cytokines with MMP-9 in COPD pathomechanism.

**Figure 3:**
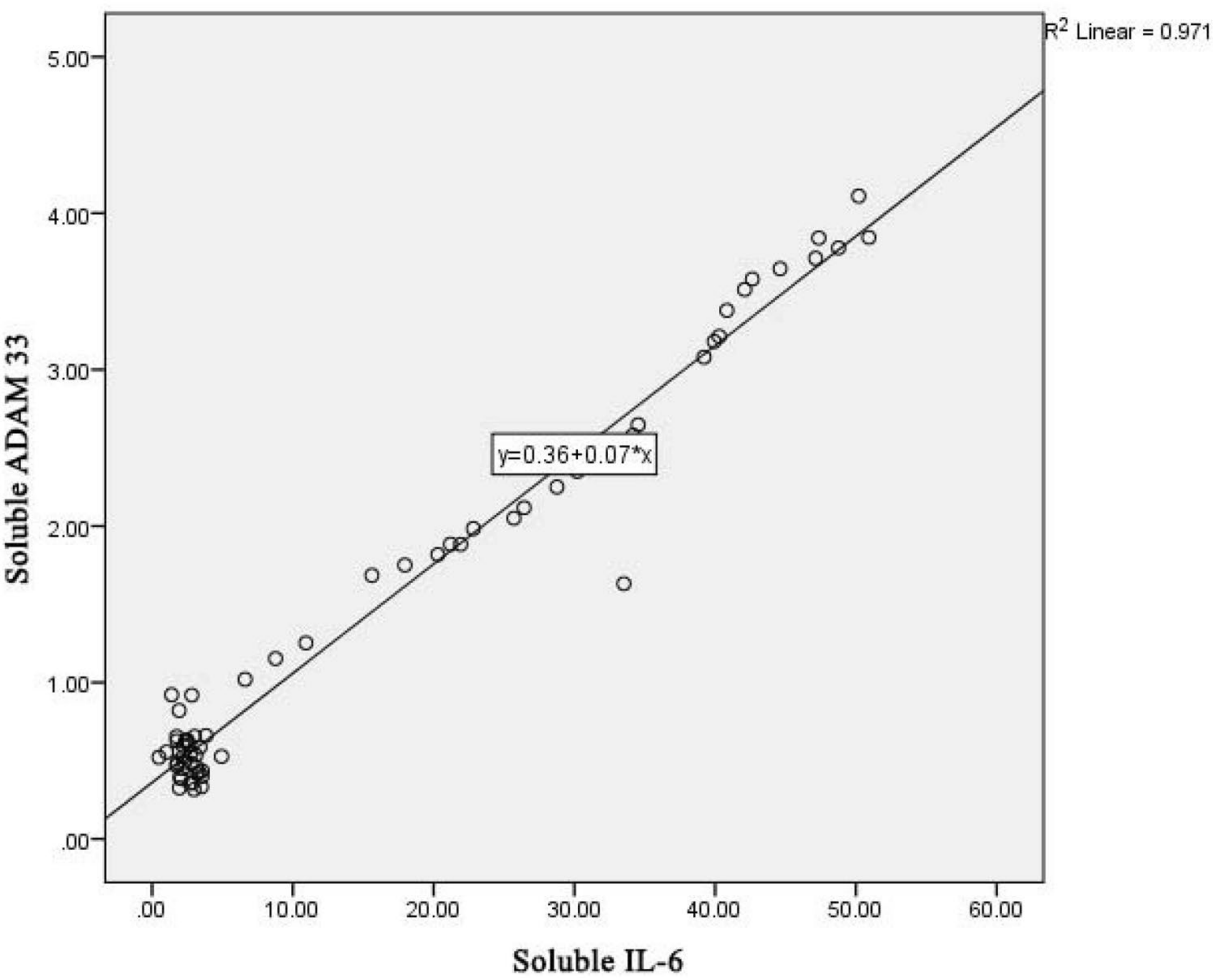
Linear regression graph between IL-6 and *ADAM33* level in COPD patients.

There is an association between *ADAM33* and inflammation in COPD patients as indicated by the correlation of *ADAM33* with MMP-9 and cytokine levels (IL-6, IL-8 and IL-10) in COPD patients. On Fig. 3 shows a linear regression graph between soluble levels of IL-6 and soluble levels of *ADAM33* with the determinant R^2^ of 0.971; it means that the contribution of soluble IL-6 levels to the soluble levels of *ADAM33* is 97.1%. The higher the soluble IL-6 level, the higher the serum soluble *ADAM33* level, which is the relationship between IL-6 cytokines and *ADAM33* in COPD pathomechanism.

**Table 7:** Correlation of soluble cytokine levels (IL-6, IL-8 and IL-10) with MMP-9.

Variable correlation	Case (COPD patient) (n = 37)		Control (not COPD patient) (n = 29)	
	r	P*	R	P*
Soluble IL-6 with soluble MMP-9	0.973	< 0.001	− 0.003	0.494
Soluble IL-8 with soluble MMP-9	0.974	< 0.001	0.497	0.003
Soluble IL-10 with soluble MMP-9	− 0.943	< 0.001	− 0.467	0.005

*Spearman correlation.

**Figure 4:**
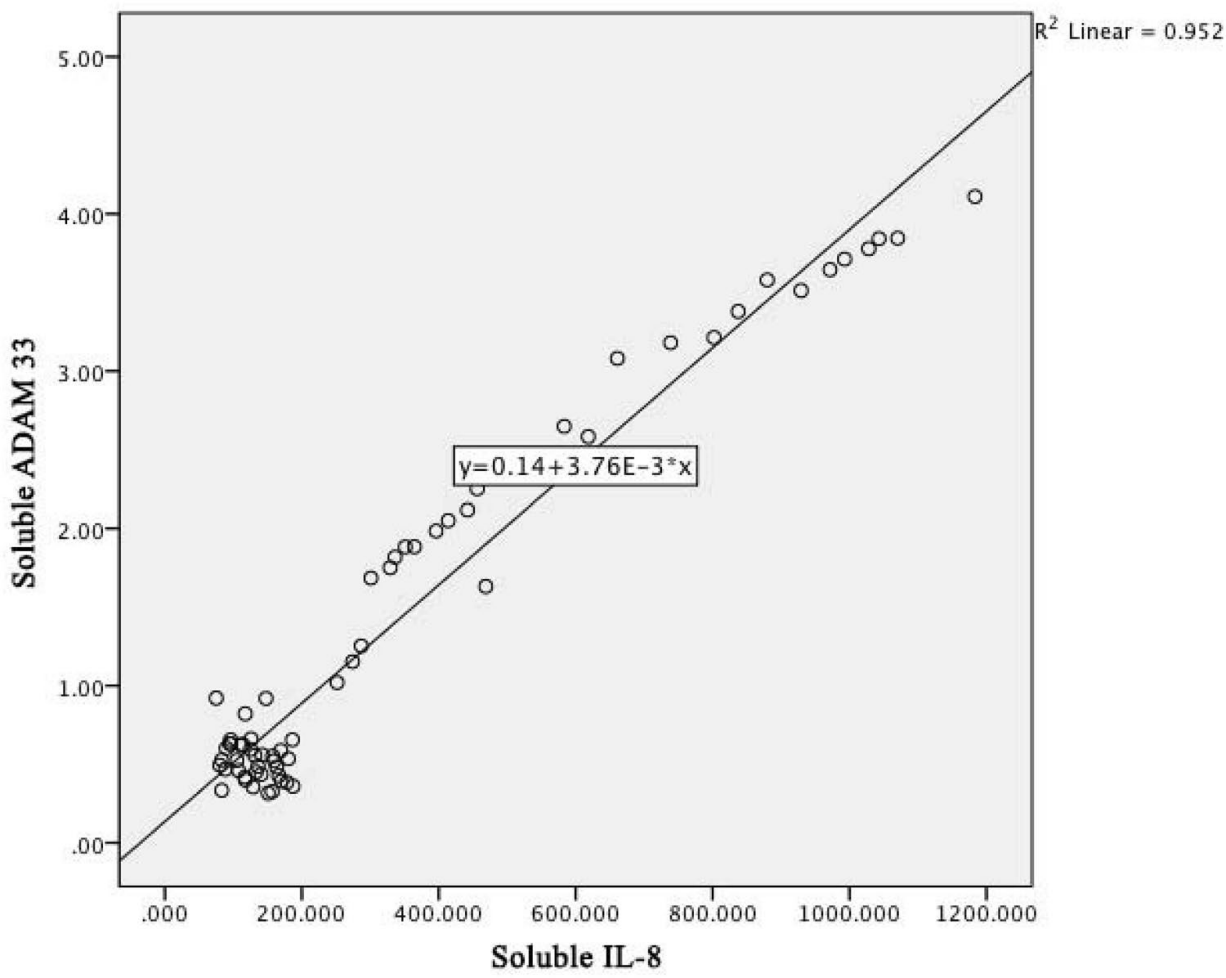
Linear regression graph between IL-8 and *ADAM33* level in COPD patients.

On Fig. 4 shows a linear regression graph between soluble levels of IL-8 and soluble levels of *ADAM33* with the determinant R^2^ of 0.952; it means that the contribution of soluble IL-8 levels to the soluble levels of *ADAM33* is 95.2%. The higher the soluble IL-8 level, the higher the serum soluble *ADAM33* level, which is the relationship between IL-8 cytokines and *ADAM33* in COPD pathomechanism.

**Figure 5:**
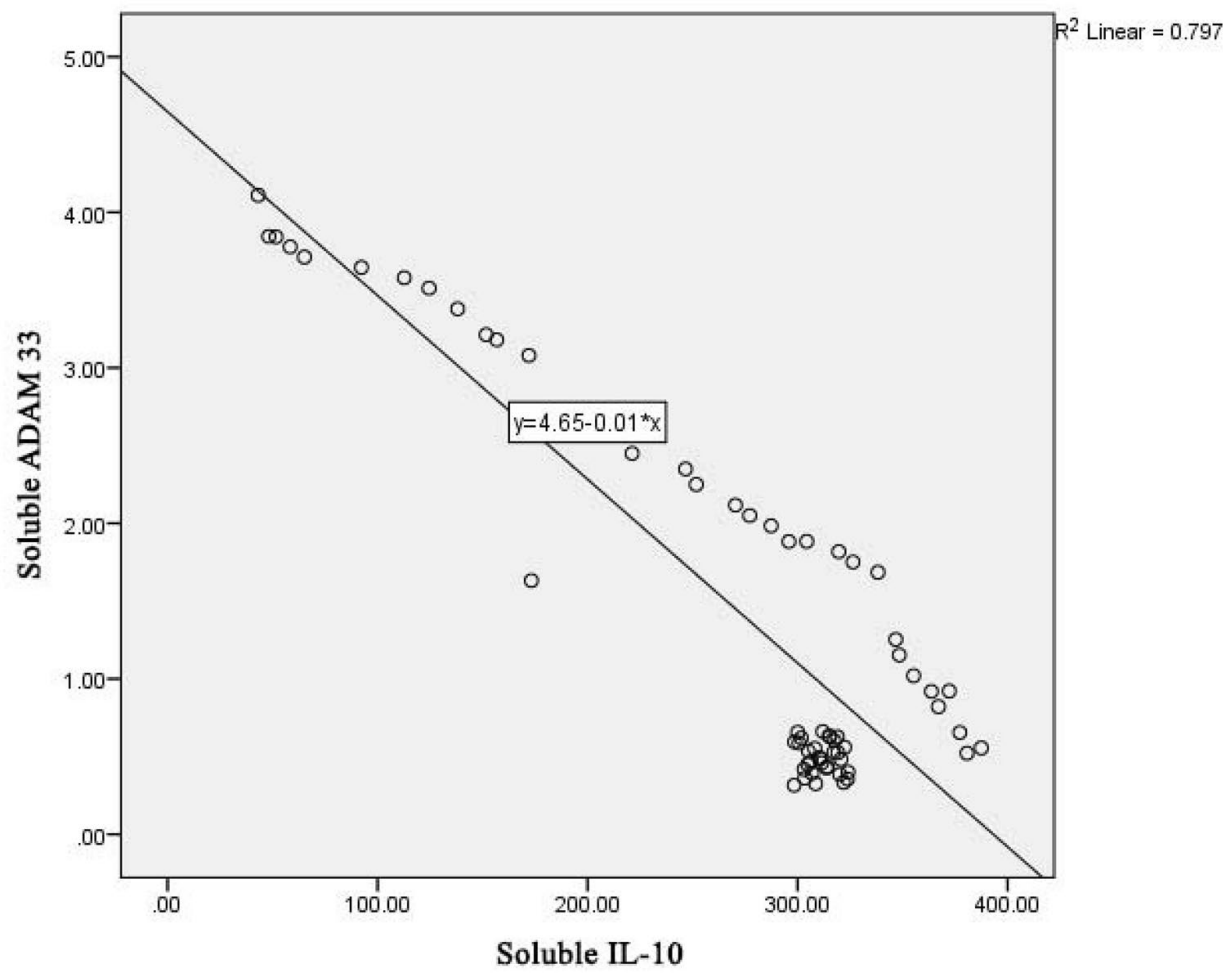
Linear regression graph between IL-10 and *ADAM33* level in COPD patients.

Figure 5 shows a linear regression graph between soluble levels of IL-10 and soluble levels of *ADAM33* with the determinant R^2^ of 0.797; it means that the contribution of soluble IL-10 levels to the soluble levels of *ADAM33* is 79.7%. The lower the soluble IL-10 level, the higher the serum soluble *ADAM33* level, which is the relationship between IL-10 cytokines and *ADAM33* in COPD pathomechanism.

**Figure 6:**
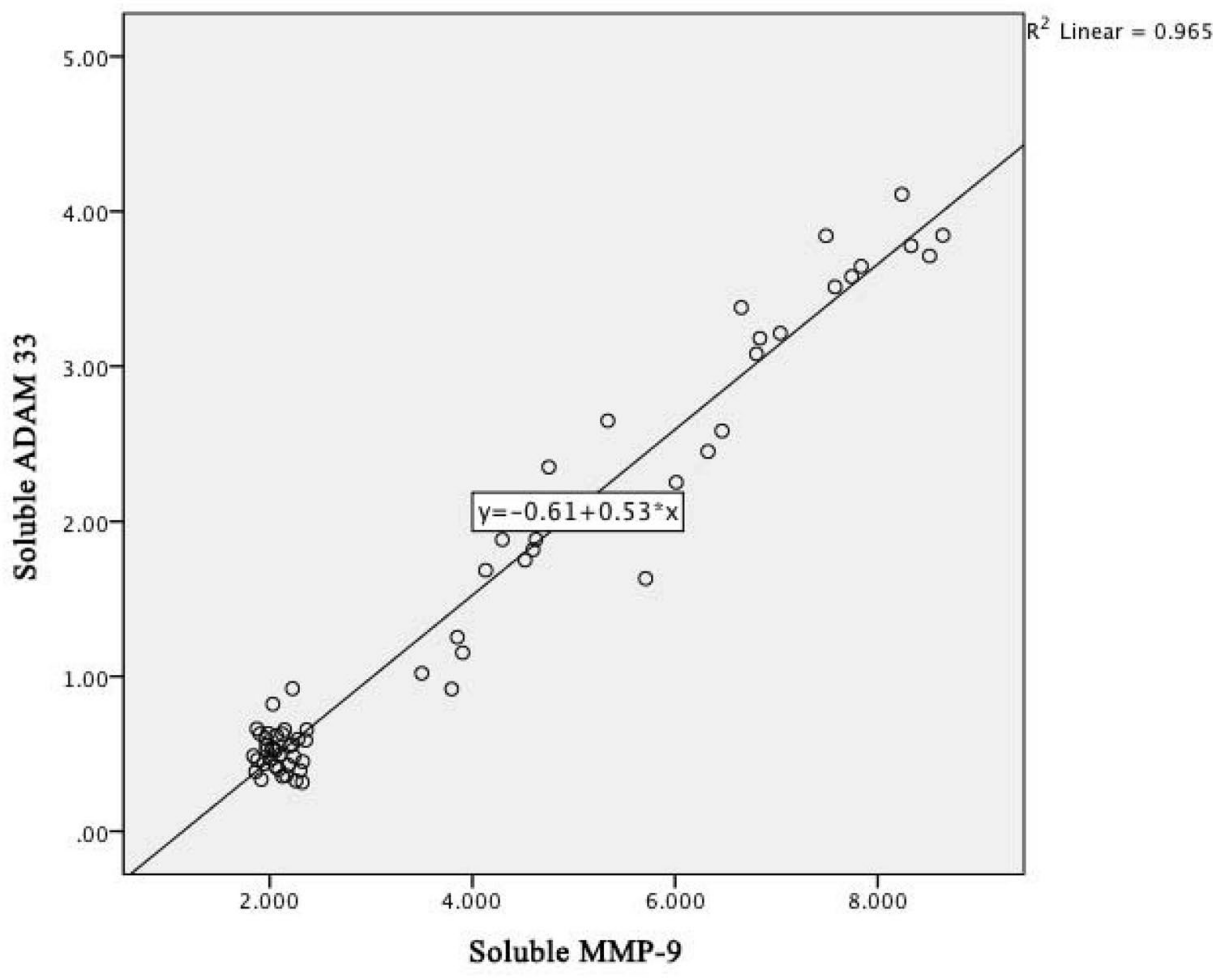
Linear regression graph between MMP-9 and ADAM33 level in COPD patients.

Figure 6 below shows a linear regression graph between the soluble levels of MMP-9 and the soluble levels of *ADAM33* with the determinant R^2^ of 0.965; it means that the contribution of the soluble content of MMP-9 to the soluble content of *ADAM33* is 96.5%. The higher the soluble MMP-9 level, the higher the serum soluble *ADAM33* level, which is the relationship between MMP-9 cytokines and *ADAM33* in COPD pathomechanism.

**Figure 7:**
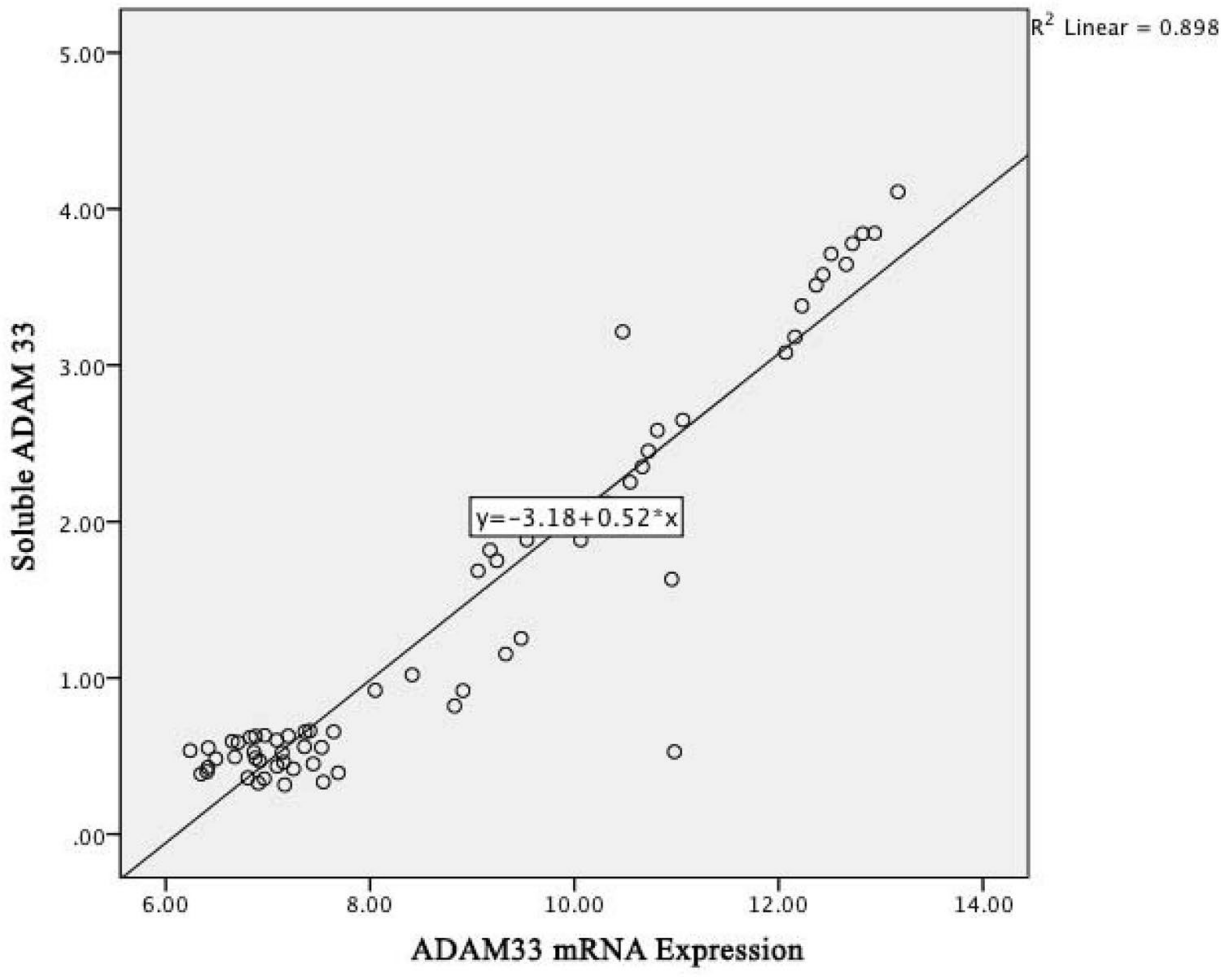
Linear regression graph between of *ADAM33* mRNA expression and *ADAM33* soluble levels in COPD patients.

Figure 7 below shows a linear regression graph between *ADAM33* gene mRNA expression and *ADAM33* soluble content with a R^2^ determinant of 0.898; it means that the contribution of *ADAM33* gene mRNA expression to *ADAM33* soluble content is 89.8%. The higher the *ADAM33* gene mRNA expression, the higher the serum ADAM33 soluble level which is the relationship between *ADAM33* gene mRNA expression and *ADAM33* soluble levels in COPD pathomechanism.

Table 8 shows the correlation of ADAM33 level between COPD and non-COPD patients according to GOLD COPD stage and their inter GOLD COPD stage. ADAM33 levels at each level of GOLD COPD stage have a high deviation value as well as ADAM33 levels in non-COPD patients, so that if ADAM33 levels at each level of GOLD COPD stage, the significance value is low. This is the same as the relationship between ADAM33 levels of inter-GOLD COPD stage, except for the statistically significant correlation of ADAM33 levels in GOLD COPD stage II and IV.

**Table 8:**
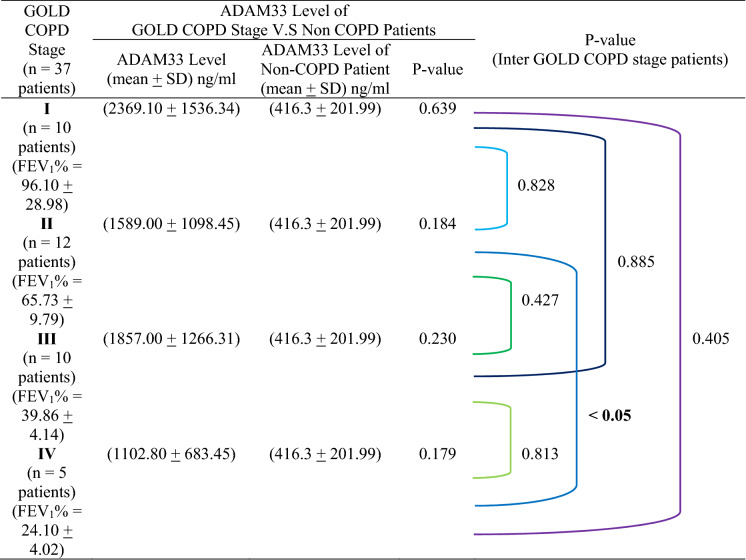
The correlation of ADAM33 level between COPD and non-COPD patients according to GOLD COPD stage and their inter GOLD COPD stage.

**Figure 8:**
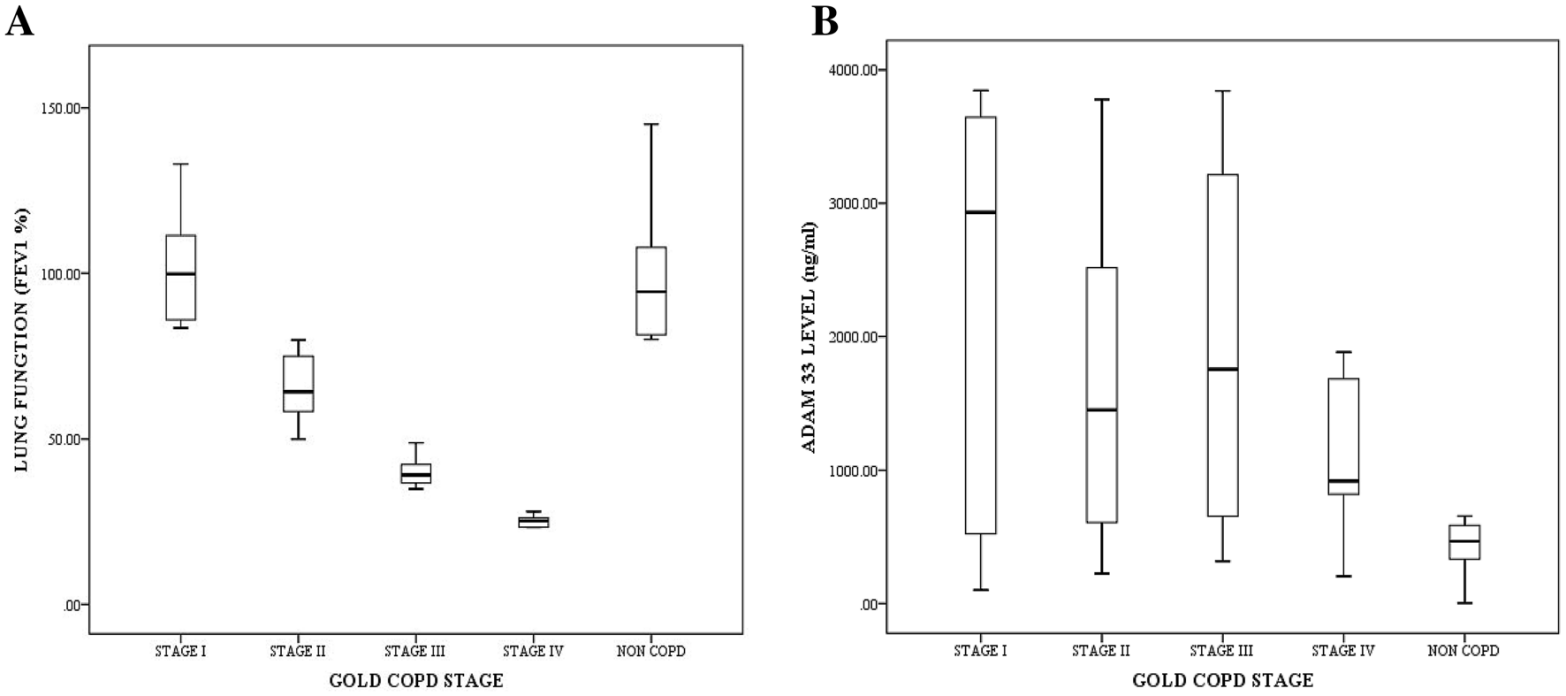
The correlation between lung function, ADAM33 level and GOLD COPD stage in COPD and non-COPD patients; (**A**) the lung function and GOLD COPD stage in COPD and non-COPD patient; (**B**) the ADAM33 level and GOLD COPD stage in COPD and non-COPD patients.

In Fig. 8 shows the correlation between lung function, ADAM33 and GOLD COPD stage in COPD and non-COPD patients. In Fig. 8A the lung function (FEV_1_%) and GOLD COPD stage in COPD and non-COPD patients. The mean value of lung function (FEV_1_%) decreases according to a range of lung function values (FEV_1_%) of GOLD COPD stage I to IV, except for GOLD COPD stage II the tendency for the average lung function value (FEV_1_%) to approach the lowest limit of the range of lung function values (FEV_1_%) of GOLD COPD stage II. In GOLD COPD stage III, the mean value of lung function tends to approach the highest limit of the range of lung function values (FEV_1_%) of GOLD COPD stage III. In Fig. 8B shows ADAM33 levels (ng/ml) and GOLD COPD stage I to IV in COPD and non-COPD patients tend to be lower, except for the average ADAM33 levels in GOLD COPD stage II which tends to be lower when associated with the average ADAM33 levels in GOLD COPD stage III.

## Discussion

The results of this study were in accordance with those reported by Shamara and Fachri (2014): majority of COPD patients were stable (37 people, 86%) and 60–69 years (16 people, 37.2%). Moreover, 19 stable COPD patients (44.2%) each manifested with a moderate (2) or severe (3) Brinkmann Index owing to cigarette smoking^15,16^. The results of the present study were also in accordance with those seen in COPD patients treated in the Department of Pulmonology, Faculty of Medicine, Persahabatan Hospital, Indonesia: majority of the COPD patients were males (86.2%) and this can be attributed to the difference in the percentage of male and female smokers^17^. Similarly, Suradi et al. showed that the prevalence of COPD was greater in men than in women comprising a cohort of 49 men and 16 women of which 47 men (72%) were smokers with acute exacerbation of COPD and positive sputum cultures of Mycobacterium tuberculosis in both DM and without type 2 DM^18–21^]. The present study also revealed a role for *ADAM33* as a biomarker of inflammation in COPD patients. We found higher levels of *ADAM33* mRNA and soluble *ADAM33* in COPD patients than that in non-COPD individuals (P < 0.01).

Among the numerous studies that have examined the role of *ADAM33* mRNA and soluble *ADAM33* levels, most are limited to patients with asthma. Foley et al. showed that the mRNA levels of *ADAM33* in in vitro cultured primary bronchial epithelial cells from asthma patients were higher than that in donor epithelial cells from asthma patients and cells from normal individuals^22^. Ito et al. compared *ADAM33* mRNA levels in smooth muscle cells from asthma patients to that in controls by determining the percentage of the total smooth muscle cells both groups; the ratio of positive/total smooth muscle cells was higher in patients with asthma than that in control individuals. The mRNA levels of *ADAM33* were higher in smooth muscle cells from patients with asthma than that in control individuals (P = 0.002)^23^. Puxeddu et al. demonstrated an increase in the levels of soluble *ADAM33* and its role in angiogenesis; *ADAM33* acts as a remodeling gene that functions independent of airway inflammation in airway obstruction through an inflammatory mechanism. Thus, *ADAM33* contributes to the pathogenesis of asthma and COPD by interactions between genetic and environmental factors^24^. Taken together, to the best of our knowledge, this is the first report of the mRNA and soluble *ADAM33* levels in COPD patients and control individuals.

In the study of Moraes et al., there was agreement with this study, the relationship between biomarkers of the inflammatory reaction of COPD patients and the control group, indicated by higher soluble IL-6 levels in the COPD patient group (32.97 ± 31.08 pg/ml) than the control group. control (5.42 ± 3.72 pg/ml; P = 0.0110). And indicated by higher soluble IL-8 levels in COPD patients (23.63 ± 14.58 pg/ml) than in the control group (13.05 ± 6.73 pg/ml; P = 0.0217)^25^. Soluble IL-10 levels of COPD GOLD I (mean = 8.5 ± 2.7 pg/mg tissue) and COPD GOLD II patients (mean = 7.8 ± 1.8 pg/mg tissue) were actually higher lower than patients with normal lung function (mean = 17.9 ± 3.1 pg/mg tissue, P < 0.05). Hackett et al.'s study is in accordance with this study, namely COPD patients with mild to moderate airflow obstruction (COPD GOLD I and II) levels of soluble IL-10 as an anti-inflammatory cytokine were lower in COPD patients than non-COPD patients^26^.

In a review article by Katarzyna Grzela et al. based on some research from Cataldo et al. 2002, Gagliardo et al. 2009, Lee et al. 2001 and Lem-jabbar et al. 1999, there were elevated levels of MMP-9 found in serum, sputum and bronchoalveolarlavage from patients with asthma exacerbations. In COPD patients, this review article is based on a study by Brajer et al. 2008 and Erlewyn-La-jeunesse et al. 2008 also showed an increase in serum MMP-9 levels, which was more negatively correlated with the Tiffeneau-Pinelli index (FEV1/FVC ratio). The level and activity of MMP-9 from sputum samples of COPD patients according to the study of Culpitt et al. 2005, found to be 12 times higher than the control group. So, the increase in serum MMP-9 levels in asthmatic patients is not as high as in COPD patients^27^. A study conducted by Jie Ji et al. revealed that serum MMP-9 levels (ng/ml) were higher in COPD patients with smokers (757 (557–1000)) and COPD patients who did not smoke (490 (382–801)) compared to controls who did not smoke. healthy (430 (251–577)) (P = 0.006). Elevated levels of MMP-9 in the extracellular matrix are essential for the remodeling process of COPD patients and their expression is regulated by specific inhibitors, such as tissue inhibitor of metalloproteinases-1 (TIMP-1). Increased TIMP-1 levels in BAL fluid from both groups of smokers compared with healthy non-smokers and increased serum levels of MMP-9 in the COPD group. Increased levels of soluble MMP-9 and TIMP-1 have been observed in serum, sputum and LAB fluid in COPD. The decrease in plasma MMP-9 levels and the inconsistent increase in TIMP-1 in COPD may be due to the fact that MMP-9 levels can vary over time in relation to the severity of COPD and smoking habits in COPD patients^28^.

In Ji et al.'s study it was shown that smokers have ongoing inflammation in the central airways (sputum), peripheral airways (BAL fluid), and systemically (blood) and that this inflammatory response is more associated with smoking than with the presence or absence of smoking. chronic airflow limitation. Although IL-8 and MMP-9 levels did not differ between the two groups, there was a significant negative relationship between salivary IL-8 and MMP-9 levels and lung function in COPD. Inflammatory markers in saliva may be associated with disease severity in COPD. A similar relationship was shown between these biomarkers in serum and lung function. This study found a very strong correlation between IL-8 and MMP-9 in saliva and periodontal inflammation as assessed by gingival bleeding in healthy non-smokers but not in the two groups of smokers. These findings suggest that these markers of inflammation in saliva are associated with periodontal inflammation under normal circumstances and this association was not seen in smokers when inflammatory activity is triggered by strong proinflammatory stimuli such as cigarette smoke^28^. Elevated levels of MMP-9 in the extracellular matrix are important for the remodeling process in COPD and its expression is thought to be regulated by specific inhibitors, such as TIMP-1. Ji et al. found increased levels of TIMP-1 in BAL fluid from both groups of smokers compared with nonsmokers and increased levels of MMP-9 in serum in the COPD group. Elevated levels of MMP-9 and TIMP-1 have been observed in serum, sputum and BAL fluid in COPD patients. However, there are conflicting results showing decreased plasma MMP-9 and TIMP-1 levels in COPD. These inconsistent results may be because MMP levels can vary over time in COPD. Differences in disease severity and smoking habits in the study population may also explain differences in results between studies^28^.

Soluble *ADAM33* and MMPs are important enzymes in processes involved in remodeling and degradation of the extracellular matrix. Increased MMP-9 activity is an important part of the progression of COPD patients, associated with other components in the pathogenesis of COPD. Thus *ADAM33* is responsible for airway remodeling and bronchial hyperresponsiveness in the early years of life. The genetic heterogeneity found in this study suggests that asthma is caused by multiple mutations in the same gene. On the other hand, different patterns of linkage disequilibrium (LD) among different populations reveal that the unprocessed causative factors may be the same, but the assortment of SNPs in strong LD with variance differs among ethnic groups. Future research into the genetics of asthma and COPD should evaluate the genetic background of patients with a history of asthma progressing to irreversible airway obstruction with a COPD-like phenotype. All basic science studies explaining genetic changes will have little meaning for doctors if we cannot put this knowledge to use in clinical practice. The genetic profile of each individual and the consequent pattern of enzymatic expression can lead us to establish a rapid diagnosis of potential severe asthma, airway improvement and the gradual and rapid establishment of COPD cases, in smokers and no smokers. It is well known that subjects with lower impairment in pulmonary function or with normal limits on function tests exhibit a greater risk of death and the need for hospitalization. This will benefit all candidate patients and healthcare providers^29^.

The research of Fei Xu et al., in accordance with this study, can prove that in chronic inflammation there is damage and metaplasia of the respiratory epithelium. Inflammation that occurs in a long time causes the formation of connective tissue in the walls of the airways. The airway wall epithelium produces epidermal growth factors (EGFs/epidermal growth factors) and various protease enzymes, especially MMP-9, which inhibits the degradation of the extracellular matrix so that changes in composition and quantity are associated with thickening of the airway wall epithelium. Smoking habits inhale many chemical compounds that induce chronic inflammation and damage to the airways. Chronic inflammation contributes to structural and cellular damage to the airway wall epithelium, resulting in fibrosis and damage to small airways. Cytokines on airway inflammation in COPD patients, such as IL-6, IL-8 as an increased proinflammatory cytokine and IL-10 as a decreased anti-inflammatory cytokine. Smoking habits cause endothelial cell damage and dysfunction, deposition of the extracellular matrix occurs where the matrix protein profile has changed, resulting in airway remodelling. So in the airways of COPD patients who have undergone remodelling there is an increase in MMP-9^30^.

As members of the zinc-dependent ADAM-metalloproteinase superfamily, *ADAM33* regulate their own function and that of a variety of other proteins by proteolytic cleavage. *ADAM33* has multiple domains including prodomain, catalytic, metalloprotease, disintegrin (binding integrin), cysteine-rich/epidermal growth factor (cell–cell contact), transmembrane and cytoplasmic domains, and has multiple forms containing various combinations of these domains. *ADAM33* is predominantly expressed in airway structural cells, including airway epithelium, airway smooth muscle, myofibroblasts and fibroblasts, thought to have a broad spectrum of functions, such as protease dependent and independent mechanisms. *ADAM33* cleaves proteins from the cell surface and may facilitate the release of cytokines and growth factors. The pathogenesis of COPD involves recruitment and regulation of neutrophils, macrophages and lymphocytes to the lung, as well as an induced imbalance between proteinases and antiproteinases, all of which result in lung parenchymal destruction and airway remodelling. Based on the expression profile and function, *ADAM33* is involved in the pathogenesis of the COPD. The study of Xinyan Wang et al. showed that the (single nucleotide polymorphisms) SNP Q-1 in the *ADAM33* gene was statistically significant (P > 0.01) associated with the release of IL-8 in sputum samples from COPD patients. Therefore, *ADAM33* can affect the release of cytokines and growth factors, causing inflammatory cell infiltration in the airways^3^.

In the data of this study, the correlation of the mean lung function and the GOLD COPD stage and non-COPD as shown by the boxplot Fig. 8A diagram, there is a tendency for the GOLD COPD stage II to approach the lowest limit of the lung function value range (FEV_1_%) GOLD COPD stage II, whereas in GOLD COPD stage III the mean lung function value (FEV_1_%) tends to approach the highest limit of the lung function range (FEV1%) of GOLD COPD stage III. In the correlation level of ADAM33 as shown in the boxplot diagram Fig. 8B, there is an average ADAM33 level in GOLD COPD stage II which tends to be lower when associated with an average ADAM33 level in GOLD COPD stage III. This is because in GOLD COPD stage II the tendency of the lung function average value to approach the lowest limit of the lung function value range (FEV_1_%) GOLD COPD stage II, while in GOLD COPD stage III the lung function average value tends to approach the highest limit of the lung function value range (FEV_1_%) GOLD COPD stage III.

The fibrotic extent of lung tissue is not directly related to oxygen consumption. The lung function (FEV1%) and *ADAM33* level not directly related because *ADAM33* has involved in fibrotic of lung tissue mechanism, however the lung function determined by oxygen level. Thus, we did not find directly the relation between *ADAM33* level and Lung Function (FEV1%)^3,23,24,29,30^.

## Conclusion

COPD patients showed significantly higher mRNA and soluble *ADAM33* levels as compared to that in non-COPD individuals. Thus, *ADAM33* may serve as an inflammatory biomarker in COPD patients.

## Data Availability

All data generated or analyzed during this study are included in this published article.
